# Treatment effects on compulsive exercise and physical activity in eating disorders

**DOI:** 10.1186/s40337-018-0215-1

**Published:** 2018-12-13

**Authors:** Therese Fostervold Mathisen, Solfrid Bratland-Sanda, Jan H. Rosenvinge, Oddgeir Friborg, Gunn Pettersen, Kari Anne Vrabel, Jorunn Sundgot-Borgen

**Affiliations:** 1 Department of Sports Medicine, Norwegian School of Sport Sciences, Oslo, Norway; 2 Department of Sports and Physical Education and Outdoor Sciences, University of South-Eastern Norway, Bø in Telemark, Norway; 3 Department of Psychology, Faculty of Health Sciences, UiT -The Arctic University of Norway, Tromsø, Norway; 4 Research Institute of Modum Bad, Vikersund, Norway; 5 Department of Health and Care Sciences, Faculty of Health Sciences, The Arctic University of Norway, Tromsø, Norway

**Keywords:** Excessive exercise, Driven exercise, MVPA, Bulimia nervosa, Binge-eating disorder, Cognitive behaviour therapy

## Abstract

**Background:**

Dysfunctional thoughts- and use of physical activity (PA) are core symptoms of the eating disorders (ED) bulimia nervosa (BN) and binge eating disorder (BED). The compulsive desire for PA complicates a favourable treatment outcome; hence, regular, adapted PA led by personnel with competence in exercise science is rarely part of treatment of BN and BED. The present study compared cognitive behaviour therapy (CBT) with a new treatment combining physical exercise and dietary therapy (PED-t) with respect to the short- and long-term changes in the level of compulsive exercise and actual level of PA in women with BN or BED.

**Methods:**

We enrolled 187 women with BN or BED, aged 18–40 years, with BMI 17.5–35, in an outpatient randomised controlled therapy trial. Participants were randomised to PED-t or CBT, while waitlist participants served as a control group during the treatment period. The treatment covered 16 weeks, with 6- and 12 months follow-up, and outcomes included self-reported compulsive exercise (CE) and objectively measured PA, analysed by linear mixed regression models.

**Results:**

Both CBT and PED-t reduced CE from baseline (*P* < 0.01, Hedges *g* ~ 0.4), but with no difference to control group. Compared to baseline, only PED-t significantly reduced the number of patients who scored above cut-off rating for CE, but with no between-group differences. The proportion of participants complying with the official recommendation for PA neither changed following treatment, nor emerged different between the treatment arms.

**Conclusion:**

Both therapies resulted in significant improvements in compulsive exercise, a change not found in the control group, however there were no between-group differences. The findings are tempered by the low statistical power due to a small control group size. The number of participants complying with the recommendation for PA were stable throughout the study, and no change in total PA was found. Presence and intensity of CE decline with treatment, but a need to increase PA towards healthy levels remains unsolved.

**Trial registration:**

Approved by the Norwegian Regional Committee for Medical and Health Research Ethics (ID: 2013/1871, 16th of December 2013); registered in Clinical Trials (ID: NCT02079935, 17th of February 2014).

## Plain English summary

Persons with eating disorders (ED) are often described as being excessively physical active or having strong drive for physical activity (PA) to comply with unrealistic thin body ideals. This dysfunctional behaviour relates to worse treatment outcome and higher risk of relapse, and this is why PA is often restricted in treatment of ED. Such restrictions may deprive patients from the potential benefits of adapted, supervised PA. These benefits may be particularly relevant for patients with bulimia nervosa (BN) or binge eating disorder (BED) because of impaired physical health.

We studied the treatment effect on compulsive exercise and levels of PA by comparing a new therapy for women with BN or BED combining PA and dietary therapy (PED-t), to the recommended cognitive behaviour therapy (CBT) and to a control group. During the treatment and follow-ups both the PED-t and the CBT treatments reduced the frequency of compulsive exercise and produced stable levels of PA. PED-t did not trigger increased volumes of exercise. Less than 50% of women with BN or BED complied with the minimal recommendation for PA, and neither therapy resulted in any such improvements. Therefore, there is still a need to search for methods to improve this insufficient level of healthy PA.

## Background

Regular physical activity (PA) may improve mental and physical health [1, 2], and for this reason, regular PA is encouraged as a first or concurrent intervention for a range of physical- and mental illnesses [3]. However, practising regular PA has rarely been part of treatment of BN and BED [4–6], because of the excessive and compulsive exercise reported in about 20–80% of patients with eating disorders (EDs) [7–10]. Excessive or compulsive physical exercise is one of the compensatory symptoms of bulimia nervosa (BN), and described in the diagnostic criteria as being “excessive and recurrent” in nature [11]. However, several studies [7–9] indicate that the compulsion is not restricted to a matter of volume of activity. Compulsive exercise (CE) includes the maintenance of rigid exercise regimens, exercising despite physical injuries, prioritising exercise before other important activities, feelings of anxiety if unable to exercise, or rigidly imposing exercise regimens before meals, or after binge eating [12, 13]. The purpose of CE is to compensate for energy surplus notably due to binge eating, but may also serve to regulate negative affects that may appear because of the ED [9]. Contrary to BN, a diagnosis of BED does not include an “overvaluation of controlling body weight or figure” [11]. However, with the high prevalence of obesity in BED [14, 15], it has been found that individuals with BED do have concerns about their body weight, figure and appearance [16] that may trigger CE.

Low levels of PA are commonly reported among persons with BED [14, 17, 18]. For BN, the mixed findings with respect to whether persons are highly active or insufficient active, may be due to methodological differences across studies [14, 19, 20]. In general, and compared to the official recommendation for PA by the American College of Sport Medicine [21], insufficient activity levels have been reported in BN when using objective measures of activity [14, 20]. By relying on these objective measures, and the previous findings of cognitive and motivational facets [7–9], an exercise paradox appears. This paradox is caused by the presence of dysfunctional attitudes and cognitions related to exercise, indicating high levels of PA, however, with insufficient levels of objectively assessed PA.

Cognitive behavioural therapy (CBT) is the preferred treatment approach for BN and BED [22]. Here, stimulus-control techniques are used to normalise eating patterns, and a focus on changing underlying cognitions serves the purpose of undermining the psychological, maintaining factors for the ED. CBT also addresses compensatory exercise, but there is no specific evidence supporting whether CBT is effective in alleviating CE.

Re-establishing practical experience with healthy exercise, along with enhancing the theoretical understanding of exercise physiology may induce a more positive attitude towards exercise [4]. Some studies [17, 23–27] report on interventions adding structured physical exercise to treatment of BN or BED, but only one study has reported on pre-post effects on attitudes towards exercise [28]. Here, a reduction of dysfunctional attitudes towards exercise was found after completing a supervised inpatient treatment programme consisting of varied, low impact PA. However, it is unknown whether structured physical exercise interventions alter CE behaviour in a longer-term, and whether such interventions simultaneously raise the objective level of PA in persons with BN or BED.

This paper reports on the short- and long-term effects of a 16-week outpatient treatment-intervention with **p**hysical **e**xercise- and **d**ietary **t**herapy (PED-t) or CBT on compulsive exercise and objectively measured PA in women with BN or BED [29]. We aimed to test the following hypotheses:

1. Compared to a non-randomised wait-list condition both treatments bring about similar reductions in CE at post-test and at follow-up.
2. At the post-tests, more PED-t patients than in the CBT conditions will show an increased level of PA, compatible with the official recommendation by the American College of Sport Medicine.

The second hypothesis reflects the genuine nature of the PED-t, while the first one may be considered as somewhat conservative, consistent with an intention to increase the portfolio of evidence-based treatments for BN and BED, and not necessarily to demonstrate that the PED-t performs better than the CBT.

## Methods

### Participants

**Fig. 1:**
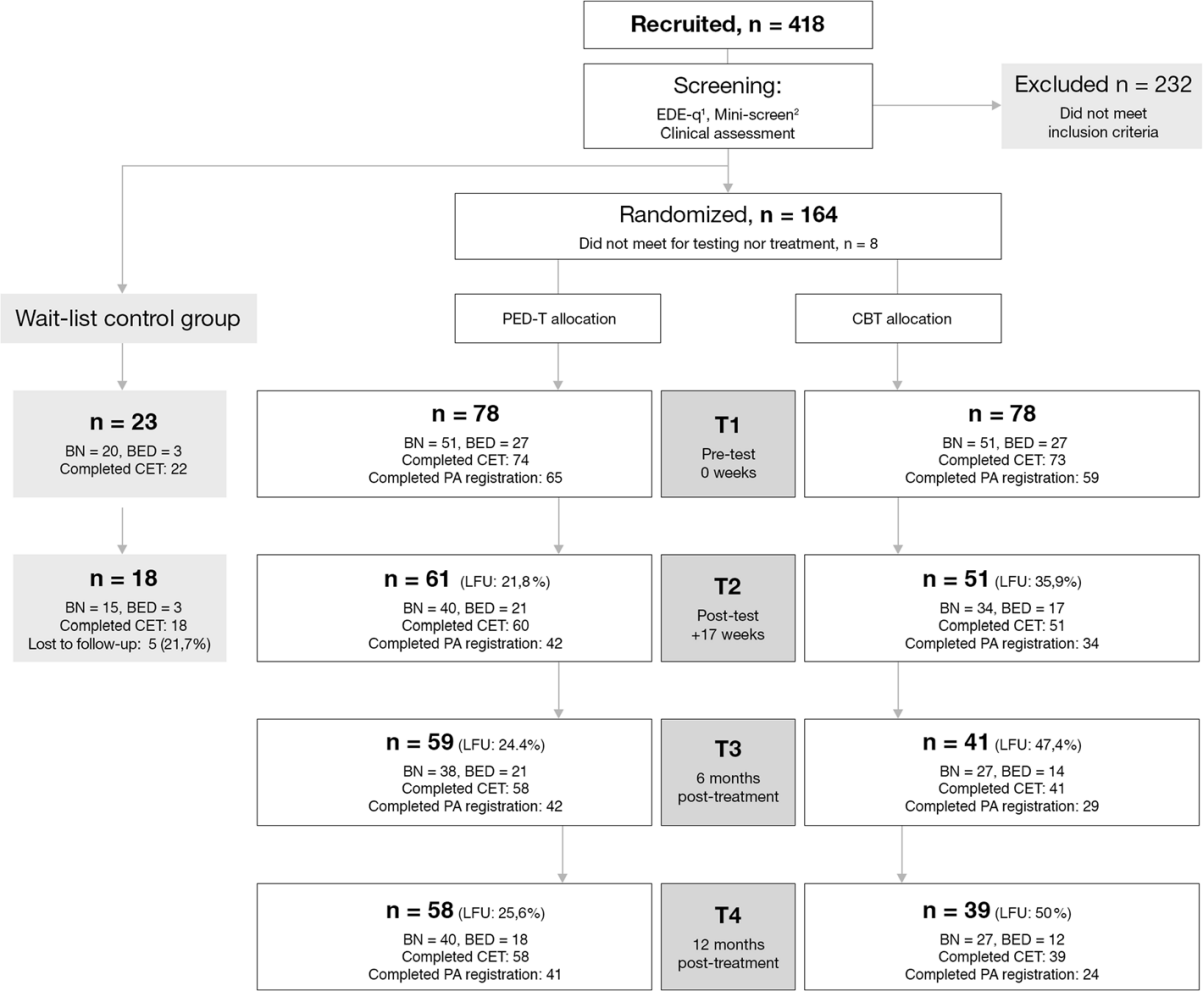
Flow chart of recruitment, screening, randomisation and attendance at pre-treatment test (T1), post-treatment test (T2) and the two follow up tests (T3 and T4). EDE-q, Eating disorder examination questionnaire; PED-t, Physical exercise and dietary therapy; CBT, cognitive behaviour therapy; BN, bulimia nervosa; BED, binge-eating disorder; CET, compulsive exercise test; PA, physical activity; LFU, lost to follow up. ^1^Fairburn et al., 2008 [30]; ^2^Sheehan et al., 1998 [42]

We recruited female participants with BN or BED for a randomised controlled trial (RCT), to investigate the effect of a new outpatient group treatment programme; the PED-t, and by comparing treatment outcomes to CBT and a non-randomised waiting list control group [29]. Participants were recruited through general practitioners (GP’s), articles and advertisement in magazines and newspapers, websites of the ED patient organisations, national TV, social media and posters. Recruitment information highlighted the CBT as the preferred therapy, still with a need to explore other treatment options as not all patients with BN or BED respond to CBT. Inclusion criteria were a DSM-5 diagnosis of BN or BED. Diagnoses were made by using the questionnaire version of the Eating Disorder Examination (EDE-q) [30] as well as a crosscheck questionnaire on behaviour according to DSM-5 diagnostic criteria. A final diagnosis was ascertained in a clinical assessment (Fig. 1). Due to the group therapy format of the PED-t and the CBT, group heterogeneity was optimised by restricting age to 18–40 years, and the BMI to range between 17.5–35.0. The BMI range was also chosen due to safety if randomised to the PED-t group; in which no individual adjustment to exercise was offered. Excluded were participants currently being pregnant, being a competitive athlete, having a concurrent severe symptom- or personality disorder in need of other treatment options, as well as those having received CBT for ED the last two years prior to the study. A final inclusion required written informed consent from the participants and their GP.

In total, 187 of recruited women were eligible for inclusion, of which 23 were temporarily allocated to a waitlist control group. The waitlist control participants were randomised to treatment groups after 16 weeks as control participants. An independent fellow co-worker assisted in creating a randomisation list (from www.randomizer.org) with block size of eight, and in a concealed allocation of the 164 recruited participants to treatment groups. Totally, 156 of the randomised participants met for baseline measures, and then successively received information on group allocation and initiated treatment. Of the included participants, we diagnosed 103 with BN and 53 with BED. In the waitlist control group, 20 were diagnosed with BN and three with BED.

### Design

The experimental design was mixed factorial as it included a between-group factor with three levels (PED-t, CBT and control), and a within-group factor with four levels (repeated assessments at baseline, post-test after 16 weeks of treatment, and two follow-ups at six and 12 months post-treatment (T1 – T4).

### Outcomes

#### Questionnaires

All participants completed the EDE-q (baseline measure in present study; Cronbach’s α = 0.87) [30], which comprises 18 items scored 0–6 to measure the presence (12 items) and the frequency (6 items) of core ED-characteristics. Cut-off scores 2.62 and 2.63 were used, which have proved valid in identifying BN and BED among Norwegian female adults [31].

Additionally, all participants completed the Compulsive Exercise Test (CET) [32] (baseline measure in present study; Cronbach’s α = 0.84). CET assesses the core features of compulsive exercise in EDs, i.e. continuance (e.g. continue to exercise despite injuries or illness), affect regulation (exercise brings about negative or positive reinforcement), weight and shape driven exercise (e.g. exercise to burn calories, or to reduce body weight), and exercise rigidity (repetitive exercise routines). CET is a 24-item instrument scored on a 5-point Likert scale (0 = never true, 5 = always true), and evaluated with five different subscale mean scores (avoidance and rule driven behaviour, weight control exercise, mood improvement, lack of exercise enjoyment, and exercise rigidity), and a global score summing the means of subscales (score range 0–30). CET has proved good internal consistency and content validity, in healthy samples and in adults with ED, with a suggested global cut-off score of 15 for indication of compulsive exercise, where higher scores indicate more compulsivity [13, 32].

#### Physical activity

Level of PA (counts/minute, CPM) were objectively measured for seven consecutive days using the ActiGraph accelerometer (ActiGraph GT3x and GT3x+, Actigraph, LCC, Pensacola, Florida, USA) placed on the right hip. Details on data recording, extraction and variable definition have been presented previously [14]. Numbers of participants measuring PA for T1-T4 are presented in Fig. 1. Reasons for not giving information on PA were; not sufficient numbers of days with registration, technical errors in separate devices, not willing to carry the device, and devices lost in mailing process.

### Treatment programmes

#### Physical exercise- and dietary therapy (PED-t)

The PED-t is a group treatment particularly designed for BN and BED by our research group, combining guided physical exercise- and dietary therapy. The exercise treatment includes theoretical sessions on exercise physiology and exercise principles, encourages to comply with the recommendations for PA (i.e. 150 min × week) [21], and adheres to recent guidelines developed from systematic reviews to successfully use therapist guided physical exercise in treatment of EDs [33]. The treatment offers 20 therapy sessions covering 16 weeks, with 1–2 weekly supervised resistance exercise session combined with a dietary therapy, and two weekly unsupervised exercise sessions (one resistance exercise and one session with high intensity interval training, respectively). All PED-t therapists had a bachelor- or master degree in exercise physiology. They were trained and introduced to the PED-t programme by the programme responsible (TFM). In addition, a dietitian was responsible for all the dietary therapy sessions.

#### Cognitive behaviour therapy (CBT)

The manual-based CBT follows a group format, and rests on a transdiagnostic model positing generic core ED-characteristics across ED-diagnoses [34]. The CBT-treatment consists of 1–2 weekly therapy sessions (20 in total) over 16 weeks of therapy. The therapy runs through four stages; 1) engagement and behavioural change, 2) monitoring and evaluating progress, 3) addressing the core pathology of ED, and 4) relapse prevention. CBT does not specifically address exercise routines other than driven exercise for compensatory reasons. Four CBT-therapists were running each of their CBT group. All four therapists had been formally trained in CBT, and had more than 10 (*n* = 2) or 20 (n = 2) years of CBT-experience in providing CBT for ED.

Details of the PED-t and the CBT treatment programmes have been published in a protocol paper elsewhere [29].

### Control group

All control group subjects fulfilled the inclusion criteria of the study. Reasons for not attending treatment immediately ranged from facing immediate longer journeys, obligations due to work/studies, living too far from treatment facilities (hence not attending therapy at all), or being recruited during an ongoing treatment period. Controls completed questionnaires at baseline (T1) and the first follow-up (T2). No data were generated at the remaining follow-ups, as all were offered randomisation into treatment groups after completing the waitlist period (i.e. after 16 weeks). No outcome from PA was available for this group.

### Statistics

All analyses were conducted in SPSS version 24 (IBM, Armonk, NY). Linear mixed regression models were built to estimate the between-group differences (PED-t vs. CBT) and the within-group changes (baseline vs. any of the three posttest measures). This analysis yields relatively unbiased estimates despite drop out given that data are missing completely at random or missing at random. Moreover, it can be safely used without conducting beforehand multiple imputations [35]. Standard errors were estimated with the restricted maximum likelihood function, and type III *F*-tests were preferred. Dependency in the outcome data was accounted for by including a random intercept factor. The fixed factors were: *Group* (0-PEDt, 1-CBT) representing the overall treatment difference, *Time* (repeated measures) representing change across measurements, and the *Group*×*Time* interaction in order to detect treatment differences at certain time points only. The between-group analyses used the baseline values as a covariate, which improves the statistical power of these tests [36]. Differences between the treatment arms were examined with planned comparisons at each time point (least square difference tests). The within-group analyses included all four measurements in the *Time* factor. Due to the number of tests, differences with *p*-values < .01 were considered as significant, and outcome data are presented as estimated means including 99% confidence intervals. Standardised Hedge’s *g* effect-sizes were calculated as a ratio of the estimated means (extracted from the mixed models) and the observed pooled standard deviations (SD). Values around 0.2, 0.5 and 0.8 were interpreted as weak, medium and strong effect sizes, respectively [37].

A comparable statistical approach was used for the dichotomous outcome variables, replacing the analysis with a generalized linear model using a binominal distribution and logit link function (reference category coded 0). Degrees of freedom were computed using Satterthwaite approximation.

We used conventional tests (i.e. *t*-, chi-square- or Fischer’s exact test to analyse dropout and loss to follow up for the two separate follow-ups). Differences with *P-*value less than 5% were accepted in these analyses.

## Results

The mean (SD) attendance rate to therapy in the PED-t treatment arm was 80.6% (11.4) and 82.1% (45.7) in CBT. In PED-t, the adherence rate to exercise sessions (supervised + unsupervised) was 69.8% for resistance exercise, and 56.7% for interval training. During the treatment period 17 (21.8%) participants in PED-t, 27 (35.9%) in the CBT and five (21.7%) in the control group dropped out. Dropouts were less physically active (g = 0.44, *P* = 0.04), and were less likely to comply with the recommendations for PA (*P* = 0.036). Compared to the PED-t group, more in the CBT group was lost to follow up at T3 (*P* = 0.026) and T4 (*P* = 0.002). Additionally, those lost to follow up at T4 were less physically active at baseline compared to the completers at T4.

There were no differences between treatment groups and control participants at T1 for age, illness duration, EDE-q global score, or diagnosis (*P* > 0.05) (Table 1).

**Table 1:** Baseline demographic and clinical information (mean (SD) or in n/percent) on participants in the PED-t, CBT and control group, respectively

Group	PED-t	CBT	Control
Age, *years*	28.2 (6.2)	27.7 (5.3)	26.5 (5.6)
Illness duration, *years*	12.9 (7.5)	12.1 (6.7)	10.6 (7.4)
EDE-q global score	3.7 (0.8)	3.7 (1.0)	3.8 (1.0)
BN, *n (%)*	49 (64.5)	48 (65.8)	20 (87.0)
BED, *n (%)*	27 (35.5)	25 (34.2)	3 (13.0)

*NOTES*: *PED-t* Physical Exercise and Dietary Therapy, *CBT* Cognitive Behavior Therapy, *EDE-q* Eating Disorder Examination Questionnaire, *BN* Bulimia nervosa, *BED* Binge-eating disorder

### Changes in compulsive exercise

#### Between group differences

**Fig. 2:**
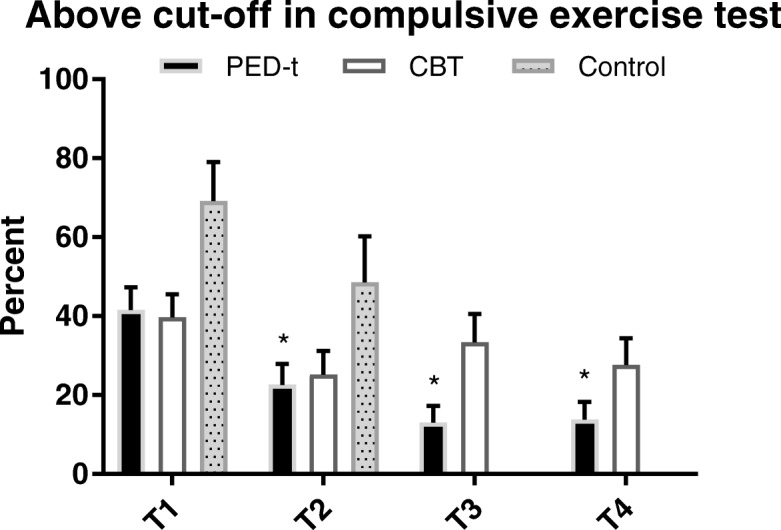
Estimated proportion (SE) of subjects scoring above the cut-off score defining compulsory exercise, across time and the treatment arms. * Significant different from baseline, P < 0.002

We found no difference between any of the three groups at any time in CET total score, subscale scores, and numbers above CET clinical cut-off (*P* > 0.01) (Table 2 and Fig. 2).

**Table 2:** Compulsive exercise test scores at baseline (T1), post-treatment (T2), 6 months (T3) and 12 months (T4) follow-up

	T1 _CI .99_	T2 _CI .99_	T3 _CI .99_	T4 _CI .99_	Within effects, p-value, Effect size (g)			Between effects, p-value, Effect size (g)		
					T2	T3	T4	T2	T3	T4
CET total										
PED-t	14.43 _13.61–15.26_	13.14 _12.27–14.02_	12.81 _11.92–13.69_	12.59 _11.70–13.47_	**< .001** *g = 0.49*	**<.001** *g = 0.63*	**<.001** *g = 0.63*			
CBT	14.21 _13.37–15.04_	13.12 _12.19–14.05_	13.27 _12.28–14.26_	12.87 _11.86–13.87_	**<.001** *g = 0.41*	**.007** *g = 0.44*	**<.001** *g = 0.61*	*n.s.*	*n.s.*	*n.s.*
CONTROL	15.18 _13.66–16.68_	14.63 _13.02–16.24_	–	–	*n.s.*			*n.s.*		
CET avoidance										
PED-t	2.34 _1.99–2.69_	1.84 _1.47–2.21_	1.73 _1.36–2.11_	1.62 _1.25–1.99_	**<.001** *g = 0.28*	**<.001** *g = 0.33*	**<.001** *g = 0.39*			
CBT	2.72 _1.92–2.63_	1.89 _1.50–2.69_	1.88 _1.47–2.30_	1.75 _1.33–2.17_	**.003** *g = 0.24*	**.005** *g = 0.25*	**<.001** *g = 0.41*	*n.s.*	*n.s.*	*n.s.*
CONTROL	2.72 _2.23–3.20_	2.56 _1.88–3.24_	–	–	*n.s.*			*n.s.*		
CET weight control										
PED-t	3.32 3.04–3.61	2.57 _2.26–.2.87_	2.71 _2.40–3.02_	2.64 _2.33–2.95_	**<.001** *g = 0.57*	**<.001** *g = 0.41*	**<.001** *g = 0.49*			
CBT	3.34 3.05–3.63	2.96 _2.63–3.29_	3.09 _2.73–3.44_	2.80 _2.44–3.15_	**.002** *g = 0.33*	*n.s.*	**<.001** *g = 0.49*	*n.s.*	*n.s.*	*n.s.*
CONTROL	3.66 3.26–4.05	3.31 _2.75–3.87_	–	–	*n.s.*			*n.s.*		
CET mood										
PED-t	4.22 3.97–4.47	4.23 3.96–4.50	4.09 _3.82–4.35_	3.96 _3.69–4.22_	*n.s.*	*n.s.*	**0.008** *g = 0.30*			
CBT	4.11 3.86–4.36	4.08 _3.79–4.36_	4.01 _3.70–4.32_	4.07 _3.75–4.38_	*n.s.*	*n.s.*	*n.s.*	*n.s.*	*n.s.*	*n.s.*
CONTROL	4.23 3.77–4.68	4.27 _3.78–4.76_	–	–	*n.s.*			*n.s.*		
CET lack of enjoy.										
PED-t	1.57 1.24–1.90	1.34 _0.99–1.69_	1.43 _1.08–1.79_	1.51 _1.16–1.86_	*n.s.*	*n.s.*	*n.s.*			
CBT	1.64 1.30–1.98	1.46 _1.09–1.83_	1.69 _1.30–2.08_	1.56 _1.17–1.96_	*n.s.*	*n.s.*	*n.s.*	*n.s.*	*n.s.*	*n.s.*
CONTROL	1.34 0.73–1.95	1.41 _0.77–2.06_	–	–	*n.s.*			*n.s.*		
CET rigidity										
PED-t	2.91 2.59–3.24	3.17 _2.82–3.52_	2.86 _2.51–3.21_	2.86 _2.50–3.21_	*n.s.*	*n.s.*	*n.s.*			
CBT	2.85 2.52–3.19	2.74 _2.36–3.11_	2.60 _2.20–3.00_	2.70 _2.29–3.11_	*n.s.*	*n.s.*	*n.s.*	*n.s.*	*n.s.*	*n.s.*
CONTROL	3.23 2.63–3.82	3.10 _2.45–3.74_	–	–	*n.s.*			*n.s.*		

*NOTES: PED-t* Physical exercise and Dietary therapy, *CBT* Cognitive Behavior Therapy, *CI*_*.99*_ 99% confidence interval. Within = Change from baseline to any of the three posttests (T2, T3 and T4); Between = Difference between groups (adjusted for baseline) at any of the three posttests (T2, T3 and T4); *g,* effect size of Hedges ‘g; *n.s.*, non-significant

Significance in bold is defined when *p* < 0.01

#### Within group changes

Compared to baseline the total CET scores were significantly reduced in both treatment groups after treatment and at follow up, and with medium effect sizes in PED-t and weak to medium in CBT (Table 2). Furthermore, both treatment groups scored lower on “CET avoidance and rule driven behaviour” (small effects in both groups) and on “CET exercise for weight control” (small to medium effects) after treatment and at follow up. There were no significant changes in the control group in CET total score or any of the CET subscales (*P* > 0.1). After treatment and during follow-up, PED-t reduced the number scoring above the clinical cut-off for CET total-score compared to baseline results (Fig. 2), but with no significant change in numbers scoring above CET clinical cut-off in CBT and control group (*P* ≥ 0.01).

### Level of physical activity (CPM)

#### Between group differences

**Fig. 3:**
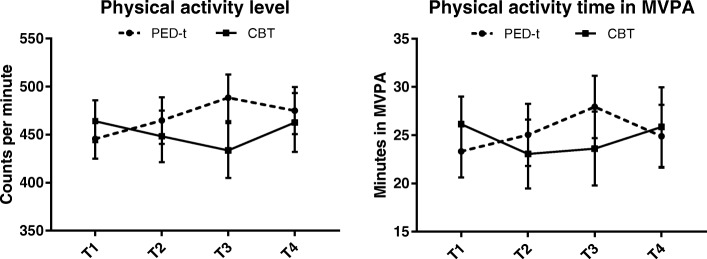
Estimated mean (SE) physical activity level (*left)* and time spent in MVPA (*right)*, from baseline (T1) to 12 month post-treatment (T4). PED-t, Physical Exercise and Dietary therapy; CBT, Cognitive Behaviour Therapy; MVPA, Moderate to Vigorous Physical Activity

No differences between groups appeared for total PA (CPM) (*P* > 0.01) nor moderate-to-vigorous physical activity (MVPA) (*P* > 0.06) (Fig. 3). We found no significant differences between the groups in the proportion complying with the recommendation for PA (*P* > 0.05).

#### Within group changes

Neither mean total PA (CPM) nor MVPA changed from baseline to any of the three post-tests in any group (*P* > 0.08 and *P* > 0.2, respectively) (Fig. 3). In PED-t, the numbers complying with the recommendation for PA varied between 45.4–56.4%, but with no significant change by time (*P* > 0.2). The corresponding results for CBT were 42.4–53.9%, also with no significant change by time (*P* > 0.5).

## Discussion

This study investigated effects of a 16-week outpatient treatment-intervention with PED-t or CBT on compulsive exercise (CE) and objectively measured PA in women with BN or BED. As hypothesised, both therapies resulted in significant improvements in compulsive exercise, a change not found in the control group, however there were no between-group differences. The findings are tempered by the low statistical power due to a small control group size.Our second hypothesis was not supported, as those participating in the PED-t treatment arm did not achieve a higher level of PA compared to the CBT participants.

Since CBT does not specifically address exercise routines other than driven exercise for compensatory reasons, there are recommendations on the development of new or adjuvant interventions to CBT, to deal with dysfunctional exercise attitudes [38]. Hence, our finding, that CBT reduced the compulsive nature of PA is encouraging considering the sparse evidence for such an effect of CBT in the literature. Also encouraging is that the PED-t reduced CE, notably the need to exercise for weight control purposes, and the need to comply with self-inflicted rules for physical exercise. In addition, there was a steady reduction in number of patients who scored above CE cut-off in PED-t. Hence, the PED-t may stand out as a promising new pathway to reduce CE. Additional benefits of inclusion of PA to treatment of EDs can be to improve the poor physical health reported in females with BN and BED (19) and to improve the overall quality of life [2]. These findings contradict arguments [39, 40] against including guided PE in treating patients with ED.

The CET total- and subscale scores in both treatment groups were lower than previously found in an inpatient sample with dispersion of diagnoses [8], but comparable to a previous finding predominately among patients with BN and BED [32]. However, in contrast to that study, we found higher mean scores on the subscale “mood improvement” and lower mean score on “lack of exercise enjoyment”, which did not change by time. These findings may indicate a sampling effect, i.e. that a high motivation for physical exercise was due to the fact that the participants had responded to treatment recruitment offering physical exercise. Hence, the results on these subscales reflect initial positive attitudes towards physical exercise despite illness severity. Moreover, the reduced level of overall CE in both intervention groups, suggest that such comparable long-term effects may be achieved through rather different therapeutic pathways and approaches.

### Level of physical exercise

Our results contradict previous findings that females with BN practice high volumes of exercise [10]. In total, only 46% were complying with the *minimal* recommendation for MVPA at baseline, concurrently, 40.8% scored above the clinical cut-off for total CET score, with high scorings not necessarily coinciding. This confirms the “exercise paradox”, i.e. that dysfunctional attitudes and cognitions related to exercise are high, whereas the actual level of PA is insufficient.

A failure to identify the reasons and motives why persons with ED patients exercise (e.g. exercise to burn calories and lose weight), may miss out important aspect of the nature of EDs, which in return could compromise the prospect of a favourable treatment outcome [33, 40]. Guidelines for using PA to treat ED amplify the need to change negative attitudes towards exercise and the exercise-related psychopathology [33]. However, these guidelines do not include recommendations on how to increase PA among persons with EDs who are inactive or active below the level of health benefits. Despite a special focus in the PED-t of regularly supervised exercise sessions and education on the health benefits of sufficient overall PA, patients in the PED-t did not increase the level PA compatible with the official recommendation by the American College of Sport Medicine [21]. Importantly though, these findings help undermine any fear of exaggerating exercise volumes if implementing exercise in the treatment of females with BN or BED.

### Strengths and limitations

High generalizability by recruiting participants with BN or BED from the general population is tempered by excluding mental disorder comorbidity. Combining psychological measures of motives for physical exercise and objective measures of exercise made it possible to study different facets of dysfunctional exercise. Objective measures of PA may reduce the risk of overestimations from self-reports [20], but they may also increase the risk of under-reporting the impact of static movement and weight lifting activities [41], which were practiced during the PED-t treatment condition. Another study strength was the RCT design, and that a concealed randomisation procedure prevented biased baseline measures. However, three limitations serve to temper the conclusions. The first one is related to the non-randomised control group and the fact that systematic differences between the controls and the other participants could bias the findings. Secondly, the statistical power was restricted due to high drop-out rate notably among the CBT participant, as well as the fact that the controls were not measured at all measure points. As a consequence of the latter, the interpretation of the long-term effect is uncertain; still, both treatments appeared to maintain their effects during the follow up period, discarding any assumption about a temporarily fluctuation in CE-behaviour. Despite the use of baseline measures as a covariate, the low power may account for the non-significant between-group differences. Finally, session-by-session data on treatment adherence and fidelity were unavailable for the current paper.

## Conclusion

Both CBT and PED-t may be viable in reducing CE in the long-term, but neither approaches raised the level of PA and compliance with official recommendations for PA.

## Abbreviations

BED: Binge-eating disorder
BN: Bulimia nervosa
CBT: Cognitive behaviour therapy
CE: Compulsive exercise
CET: Compulsive exercise test
CPM: Counts per minute
ED: Eating disorder
EDE-q: Eating disorder examination questionnaire
MVPA: Moderate to vigorous (intensity) physical activity
PA: Physical activity
PED-t: Physical activity and dietary therapy
T1: Baseline measure (Time 1)
T2: Post-test (Time 2)
T3: Follow up 6 months (Time 3)
T4: Follow up 12 months (Time 4)
